# Effects of Targeted Memory Reactivation on Cortical Networks

**DOI:** 10.3390/brainsci14020114

**Published:** 2024-01-23

**Authors:** Lorena Santamaria, Anne C. M. Koopman, Tristan Bekinschtein, Penelope Lewis

**Affiliations:** 1 School of Psychology, Cardiff University, Wales CF10 3AT, UK; santamariacovarrubiasl@cardiff.ac.uk (L.S.);; 2 School of Psychology, University of Cambridge, Cambridge CB2 1TN, UK; tb419@cam.ac.uk

**Keywords:** EEG brain connectivity, targeted memory reactivation, sleep

## Abstract

Sleep is a complex physiological process with an important role in memory consolidation characterised by a series of spatiotemporal changes in brain activity and connectivity. Here, we investigate how task-related responses differ between pre-sleep wake, sleep, and post-sleep wake. To this end, we trained participants on a serial reaction time task using both right and left hands using Targeted Memory Reactivation (TMR), in which auditory cues are associated with learned material and then re-presented in subsequent wake or sleep periods in order to elicit memory reactivation. The neural responses just after each cue showed increased theta band connectivity between frontal and other cortical regions, as well as between hemispheres, in slow wave sleep compared to pre- or post-sleep wake. This pattern was consistent across the cues associated with both right- and left-handed movements. We also searched for hand-specific connectivity and found that this could be identified in within-hemisphere connectivity after TMR cues during sleep and post-sleep sessions. The fact that we could identify which hand had been cued during sleep suggests that these connectivity measures could potentially be used to determine how successfully memory is reactivated by our manipulation. Collectively, these findings indicate that TMR modulates the brain cortical networks showing clear differences between wake and sleep connectivity patterns.

## 1. Introduction

Sleep and wake are organised into heterogeneous states that evolve continuously across time. These states are associated with clear differences in brain function and cognition. Brain connectivity provides a well-established method for studying how such states change during the transition from wake to sleep [1,2,3,4,5,6]. For instance, using non-invasive stimulation, ref. [7] reported that low-frequency oscillations such as the slow oscillations (SOs) that characterise slow wave sleep tend to travel in an antero-posterior direction. Similar findings were observed by Usami et al. when using an invasive stimulation technique called single-pulse electrical stimulation (SPES) to examine high gamma frequencies [8]. Analogous technique was used by Arbune at al. with a focus in hippocampal connectivity, reporting an increment in the number of connections to hippocampus during sleep compared with wake [9]. Brain connectivity has also been successfully used for sleep stage classification [10,11]. This includes functional brain connectivity and their physical distance nodes proximity, which have both added important spatial and topological information to the model [11].

During the transition into sleep, e.g., as the participants stopped responding to stimuli, Comsa et al. observed a breakdown of connectivity at alpha frequency accompanied by an emergence of theta connectivity [5]. Also, in the theta band, Canales et al. reported an increase in long-range information sharing (connectivity) between the brain regions during this sleep–wake transition [12]. Brain connectivity measures are not only able to reflect the differences between wake and sleep but have also proven useful in the study of how sleep changes across different ages [13], in the study of infant sleep [14], and in the identification of the relationship between specific brain regions across the sleep stages [15].

Deep non-REM sleep is strongly characterised by specific patterns of neuronal oscillation such as high amplitude SOs at ~0.8 Hz, and highly localised sleep spindles at ~10–16 Hz. Brain connectivity patterns appear to be modulated by these oscillations, and the influence of such modulation upon neural memory traces remains to be understood. A few studies have examined the relationship between brain connectivity and SOs. For instance, Boly and colleagues found that brain network integration decreased in proportion to slow wave activity [16]. Furthermore, Zhou and colleagues reported a positive correlation between dynamic connectivity states in deep non-REM sleep and slow wave activity [17]. This relationship between neural oscillations and connectivity extends to sleep spindles. For instance, in study [18], it was shown that fast sleep spindles modulate hippocampal–cortical connectivity, promoting the restructuration of memory representations. Similarly, Fang et al. reported that stronger functional connectivity during spontaneous sleep spindles predicted greater fluid intelligence [19].

Targeted memory reactivation (TMR) is a relatively recent but widely used technique to enhance memory performance by triggering memory reactivation in sleep. The TMR-induced benefits to memory were demonstrated in both animal and human studies [20,21]. In the current report, we used auditory TMR of a procedural serial reaction time task (SRTT) during slow wave sleep (SWS), a sleep stage that is strongly associated with the consolidation of this task [22,23]. Few studies have used brain connectivity to study sleep and memory reactivation in conjunction with TMR. In [24], the authors analysed the changes in sleep network dynamics in response to cued sounds versus control sounds during SWS with the aim of finding a reactivation of the linked memory trace. They found increased network integration in the occipital cortex. Schreiner et al. used phase-related similarity in theta band to study the temporal dynamics of memory reactivation for vocabulary-learning tasks that were cued during SWS using TMR [25]. While these findings support the use of brain connectivity to study the impacts of TMR on sleep and memory, gaps remain in the understanding of this phenomenon. Here, we addressed the questions related to TMR-based changes in brain connectivity across three sessions (pre-sleep, sleep, and post-sleep) during a motor imagery task, focusing not only on the differences between wake and sleep but also on the changes in pre- and post-sleep connectivity values and their relationship to behavioural performance. Additionally, we examined the feasibility of using phase-based connectivity to detect memory reactivation. We proved that TMR not only changes the functional connectivity, with clear differences between wake and deep sleep, but also that this change can be used a possible marker of how successful the memory reactivation was.

## 2. Materials and Methods

### 2.1. Experimental Task and Procedure

Participants were sixteen right-handed adults with no self-reported history of neurological, psychiatric, sleep, or motor disorders (8 females, mean age 23.1 years). All participants provided written informed consent and were reimbursed for their time. The experiment was approved by the School of Psychology, Cardiff University Research Ethics Committee. This dataset is part of a larger cohort initially presented in [26], under review, where two sleep groups were compared (SWS and REM). We focused only on the SWS whereas the REM group only has already been published in [27].

**Figure 1:**
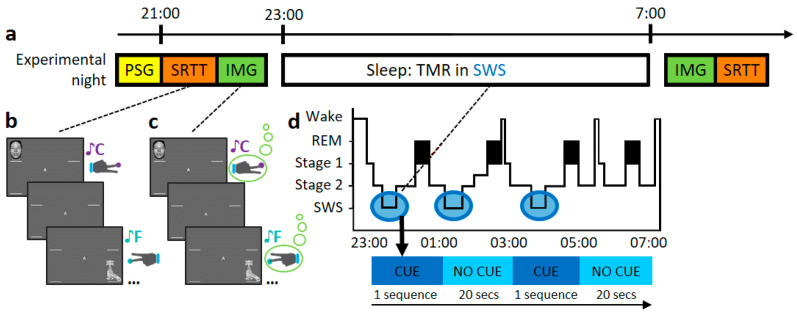
Experimental procedure. (**a**) Timeline of experimental set-up. First, participants were wired-up (EEG), after which they completed the SRTT and IMG task as outlined in (**b**,**c**), respectively. Then, participants went to sleep and TMR was carried out as described in (**d**). After waking up, participants completed the IMG and SRTT again. (**b**) Serial Reaction Time Task (SRTT). Images are presented in two different sequences. Each image is accompanied by a specific pure tone (different for each sequence) and require a specific button press. (**c**) Motor imagery task (IMG). Participants view and hear the same sequences again, but this time they are instructed to only imagine pressing the buttons. (**d**) Schematic representation of the TMR protocol. Reactivation took place in SWS (blue bubbles). One sequence was played as long as participants were in the relevant sleep stage, with a 20-s pause between repetitions. Adapted from Koopman et al. [26].

The task and experimental procedures are explained in detail in [26,27]. The outline of this study is explained in Figure 1a. As summary, participants performed an adapted serial reaction time task (SRTT) from [22] containing repeating blocks of two fixed 12-item sequences that were learned in interleaved blocks. Both sequences were matched for learning difficulty, and both contained each item three times. Participants were aware of the existence of two 12-items sequences and each sequence was indicated with ‘A’ or ‘B’ appearing centrally on the screen, but they were not asked to learn the sequence explicitly. After the 48 blocks of each sequence (A and B), participants performed 4 more blocks of semi-random sequences as a control. Each sequence was paired with a group of pure musical tones (of 200 ms duration), counterbalanced across sequences. Simultaneously to the sound, a visual cue appeared in one of the corners of the screen, where location was indicative of which key needed to be pressed (see Figure 1b). Two of the four stimuli were on the left side (top and bottom corners) indicating that left hand must be used and the other two on the right side, where participant should use the right hand. Participants were instructed to keep individual fingers of either hand on the left and right responses keys (left shift, left Ctrl and up and down arrows, respectively). Visual cues stayed on the screen until the correct key was pressed, after which an 880 ms inter-trial interval followed. Afterwards, participants were asked to do the same task again, but were instructed to only imagine pressing the buttons—corresponding to the left or right hand accordingly without performing any muscular movements (IMG, see Figure 1c). No performance feedback was presented during the pause between blocks and no random blocks were introduced for the IMG task.

Participants performed the SRTT and the motor imagery task (IMG), then had relaxation time before going to bed around 11 pm. During the night, the same tones as before were replayed in the SWS. Tones were presented as often as possible (range 978–1908), with a pause between tones of 1500 ms and with 20 s breaks between sequences (see Figure 1d). As these tones were now associated with the SRTT, we expected that replaying them should trigger the reactivation of this association (a memory) [22,23,27,28]. After 7–8 h of sleep, participants were woken up and allowed at least 20 min to overcome sleep inertia. During this time, subjects were given the opportunity to eat and drink before completing the sleep quality questionnaire. Participants then completed the same tasks again in reverse order (IMG first, SRTT second).

### 2.2. EEG Acquisition and Pre-Processing

**Figure 2:**
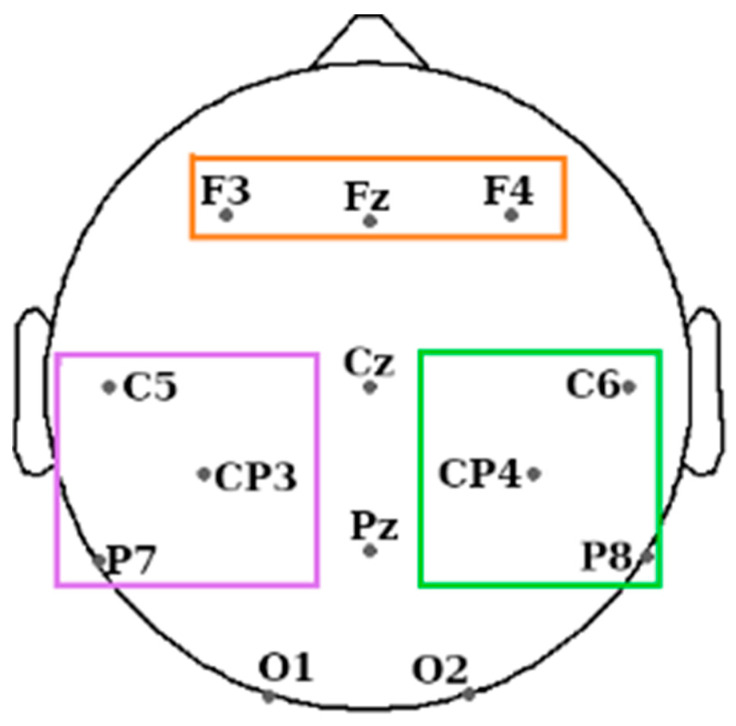
EEG electrode positions used during the experiment. Coloured rectangles indicate the three regions of interest: frontal area (orange rectangle), motor channels from the left hemisphere (LHem) in purple, and its right hemisphere (RHem) counterpart in green. The rest of the electrodes not covered by any rectangle were not used for connectivity analysis.

Twenty-one electrodes were placed on the scalp and face of the participants following the 10–20 system. On the scalp, these were at 13 standard locations: Fz, Cz, Pz, F3, F4, C5, CP3, C6, CP4, P7, P8, O1, and O2, and they were referenced to the mean of the left and right mastoid electrodes (see Figure 2). Further electrodes used were the left and right EOG, three EMG electrodes on the chin, and the ground electrode on the forehead. The impedance was <5 kΩ for each scalp electrode, and <10 kΩ for each face electrode. Recordings were made with an Embla N7000 amplifier and RemLogic 1.1 PSG Software (Natus Medical Incorporated). PSG recordings were manually scored by two trained sleep scorers according to the standard AASM criteria [29]. Both scorers were blind to the periods when the sounds were replayed.

The pre-processing was performed in Matlab^®^ and EEGLAB [30]. The continuous data were high-pass filtered at 1 Hz and low-pass filtered at 45 Hz (sync filters with 1651 and 191 points, respectively). For the sleep EEG data, trials marked as arousals or artifacts after the sleep scoring were rejected from further analysis. Eye movement and muscular artefacts were removed using independent component analysis (ICA). Bad channels were interpolated and data epoched into 2.5 s segments from −0.5 s before stimulus onset to 2 s afterwards. Finally, a visual inspection of the dataset was performed and any residuary artefact was rejected.

### 2.3. Connectivity Estimation

Phase-Locked Value (PLV) and weighted Phase Lag Index (wPLI) for all EEG channel pairs (13 × 13 pairs) were used as connectivity estimators. Connectivity was calculated using 1 s sliding window with a 50% overlap in order to obtain an idea of its temporal evolution. This gives 3 windows per trial: 0 to 1 s, 0.5 to 1.5 s, and 1 to 2 s, we will refer to them as *w*1, *w*2, and *w*3, respectively. The temporal windows were selected as the minimum size allowed to have a stable signal to calculate the connectivity, according to [31] this is between 3 and 8 cycles at a given frequency band. All computations were performed using in-house MATLAB-based functions [32].

PLV measures frequency-specific transients of phase locking independent of amplitude [31]. The instantaneous phase of the signal was calculated using the Hilbert transform, which we already used previously [32]. Two signals *x*(*t*) and *y*(*t*) with instantaneous phases $\varphi_{x}\left(t\right)\;$ and $\varphi_{y}\left(t\right)$ are considered phase synchronised if their instantaneous phase difference is constant:

(1) $$\theta \left(t\right)=\varphi_{x}\left(t\right)-\varphi_{y}\left(t\right)=constant.$$

To calculate phase synchronisation, we used PLV defined as:

(2) $$PLV=\left|\frac{1}{T}\sum_{j=1}^{T}e^{i\theta \left(t\right)}\right|$$

where *T* is the number of time samples. PLV is a value within the range [0, 1], where values close to 0 indicate random signals with unsynchronised phases and higher values indicate stronger synchronisation between the two signals (here, pairs of electrodes).

Both metrics are based on phase synchrony but focused on different aspects. Whereas PLV reflects phase synchronisation in its true sense (it looks at phase difference), PLI can be seen as the proportion of the phase difference between signals above or below 0 degrees to avoid volume conduction problems [33]. Asymmetry of this proportion of phase difference suggests the existence of time delay between the data, and, therefore, true interaction between the recorded sites [34]. While a flat distribution centred around 0 (or π, 2π…) reflects no coupling, a weighted version of the lag index (wPLI) was introduced to make those points reflecting instantaneous mixing less influential due to noisy signals [35]. This was implemented by weighting using the imaginary component of the cross-spectrum:

(3) $$wPLI=\frac{\left|\frac{1}{T}\sum_{j=1}^{T}\left|Imag\left[P_{xy}\right]sgn(Imag\left[Pxy\right]\right|\right|}{\frac{1}{T}\sum_{j=1}^{T}\left|Imag\left[P_{xy}\right]\right|}$$

where *Imag* is the imaginary part of the cross-spectral density (*P_xy_*) between the two signals *x*(*t* and *y*(*t* and *sgn* indicates sign.

Connectivity values were calculated from the theta band filtered signal (3–8 Hz). We focused on theta band because there is recent evidence suggesting potential roles of theta in NREM and REM memory processing [15,25,36]. Additionally, numerous wake studies have also investigated the theory that elevated theta oscillations during waking hours would also be associated to consolidation effects. Theta neuro-feedback training protocols led to significant improvement in performance for a motor sequence task [37,38].

Prior to calculating the connectivity values, each trial was normalised by the averaged sum of the condition 1 and condition 2, left and right hands in this case. Afterwards, the connectivity values were calculated for each trial, condition (left and right hands), window and participant for each one of the three sessions: pre-sleep (Pre), sleep (Sleep), and post-sleep (Post).

### 2.4. Statistical Analysis

#### 2.4.1. Surrogate Data Construction

To assess whether the brain connectivity values were significant above chance, a surrogate data analysis was performed which controlled from spurious (random) connections [39]. Two types of randomisations were performed, in phase and time.

For phase shuffling, a Fourier Transform was applied to each data epoch for each channel, and a random permutation of phase values was performed in the frequency domain as described in [32]. Finally, the inverse Fourier transform was used to recreate the surrogate data in time domain. This process retained the original spectral profile of the data whilst selectively disrupting phase relationships across channels, thereby removing genuine phase-based connectivity patterns. For time-shuffling, each data epoch for each channel was completely scrambled to form the surrogate data. Both obtained similar results, so here, we only present results after phase shuffling.

A total of 100 surrogate datasets were created for each participant, channel, and epoch to generate a distribution of connectivity values for the purpose of significance testing. That is, if we originally had a 16 channels × 800 trials, after the phase (or time) shuffling we will have 16 channels × 800 trials × 100 permutations. We calculated the brain connectivity of each one of these permutations as we did for the original data, creating a distribution. To perform this test, the neural connectivity indices obtained for the real data were compared against those for the surrogate data, epoch-by-epoch. The significance level of the real connectivity data was determined with reference to the surrogate distribution, meaning if the real data were greater than the 95th-centile value of the surrogate distribution was kept [34]. This comparison against the surrogate data gave us a *p*-value that was corrected for multiple comparisons using Turkey’s honestly significant difference criterion, as we did in [32]. For each epoch, the neural connections between EEG channels that were not significantly different from their respective surrogates were set to zero (and disregarded for subsequent statistical analyses of differences between conditions).

#### 2.4.2. Statistics and Reproducibility

Repeated measures ANOVA (RM-ANOVA) and its Bayesian counterpart were calculated in JASP [40] and MATLAB^®^. Further post-hoc testing was performed using Holm–Bonferroni test and paired *t*-test when applicable. In order to assure that the metrics were stable across trials, conditions, and participants, those links (of connectivity values) that were not present in 60% of the trials within participants and in 50% of trials across participants were discarded for subsequent statistical analyses [41]. Analysis of correlations and the corresponding bootstrapping corrections (*n* = 50,000) was performed in R stats and *boot* packages from CRAN repository [42,43].

### 2.5. Behavioural Analysis

The behavioural metric used for this study is the reaction time (RT) per block, differentiating between reactivated (Re) and non-reactivated (NRe) or uncued sequences, as explained in [26,27]. The averaged performances of the last four blocks before sleep were considered to represent pre-sleep ability, and this was subtracted from the random blocks to remove the effects of increased sensorimotor mapping ability. The resulting variable can thus be called sequence-specific skill (sss). Sequence-specific improvement (ssi) was then calculated for each sequence (Re and NRe) by subtracting the pre-sleep sequence-specific skill from the post-sleep sequence-specific skill as detailed below, where higher values on these metrics indicate more improvement [26].

*Re_sss_pre*: random reactivated blocks before sleep−last 4 reactivated blocks before sleep
*Re_sss_post_early*: random reactivated blocks after sleep−first 4 reactivated blocks after sleep
*Re_sss_post_late*: random reactivated blocks after sleep−last 4 reactivated blocks after sleep
*Re_ssi_early*: Re_sss_post_early−Re_sss_pre
*Re_ssi_late*: Re_sss_post_late−Re_sss_pre
Similar calculations were carried out for the NRe counterpart: NRe_sss_pre, NRe_post_early, NRe_sss_post_late, NRe_ssi_early and NRe_ssi_late.

## 3. Results

### 3.1. Behavioural Results

The differences in behavioural performance between the left and the right hands found in Koopman et al provided the motivation for the current connectivity analysis [26]. Behavioural metrics, reaction time (RT), and sequence-specific skill all presented a similar pattern where only the left-hand condition, which is the non-dominant hand in all participants, presented a TMR-related benefit. The results are outlined below, but for statistics and full details please refer to [26].

An ANOVA with the factors of time (pre- and post-sleep) and TMR (cued or uncued sequence) examined how a sequence-specific skill changed overnight for all trials (both left and right hands combined). As expected, there was a main effect on time, with faster sequence performance after sleep. There was also an interaction between the two factors, with a larger improvement for the cued compared to the uncued sequence [26].

Next, non-dominant left-hand trials and dominant right-hand trials were separated and the ANOVA described above was performed separately for each hand in order to examine the differences in how TMR affects the two hands. The left hand showed a main effect of time and time × TMR interaction. Further post-hoc analysis demonstrated a greater overnight improvement for the cued compared to the uncued sequence. By contrast, the right hand did not show any cueing-related improvement, only the time factor was statistically significant.

A complementary analysis was performed using RT instead of a sequence-specific skill (sss). Once more, when both hands were combined, the interaction between time and TMR was not significant. When separating left- and right-handed trials, there was a significant interaction effect, with the cued sequence having a greater overnight improvement than the uncued sequence.

### 3.2. Brain Connectivity

We expected differences between brain responses in the two hemispheres in trials associated with left- (L) and right-hand (R) movements while participants performed a motor imagery task. We therefore divided the 13 scalp electrodes between the right hemisphere (RHem) and the left hemisphere (LHem) (see Figure 2) and considered the “motor channels” for each hemisphere independently: C6, CP4, and P8 for the RHem and C5, CP3, and P7 for the LHem [44].

We added the frontal channels (F3, Fz, and F4) as regions of interest following the previous literature related to consciousness that reported differences in effective and functional connectivity between wake and sleep stages, in particular between frontal and parietal areas [7,45]. We calculated the connectivity between each hemisphere and the frontal channels to determine if it differed between sleep and wake. We expected differences between wake and sleep but not between L and R hands.

We excluded central channels, Cz and Pz, because we are interested in between-hemisphere differences in the potential neural markers of the motor/premotor lateralisation. Additionally, we excluded O1 and O2 so that we had the same number of channels in the three areas of interest and avoid any possible bias.

In the following sections, we present three analyses of connectivity: (1) between each hemisphere and the frontal area (LHem↔Frontal and RHem↔Frontal), (2) between hemispheres (LHem↔RHem), and (3) within hemispheres. For the first two cases, we hypothesised that no differences between hands would be found, but clear differences would emerge between wake and sleep stages. For the last case, we hypothesised that connectivity within the RHem should be larger for L-handed participants than R-handed participants and the opposite for within LHem as it is a motor imagery task. We performed each of the three analyses outlined above for each session (Pre, Sleep and Post), for each condition (R and L hands) and for each temporal window after the trial onset (*w*1: 0 to 1s, *w*2: 0.5 to 1.5s, and *w*3: 1 to 2s).

#### 3.2.1. Brain Connectivity Differences across the Sessions

Previous studies have demonstrated differences in connectivity across wake and sleep, repeatedly finding that the clear flow of information from the posterior to the frontal brain areas during wake reverses during non-REM sleep, and in REM, only the networks in the parietal regions are active [9]. Our study lacks the directionality to examine these subtle differences; however, we still expect an increment in connectivity between the frontal areas and any other brain region during sleep as compared to wake.

##### Hemisphere to Frontal Connectivity

**Figure 3:**
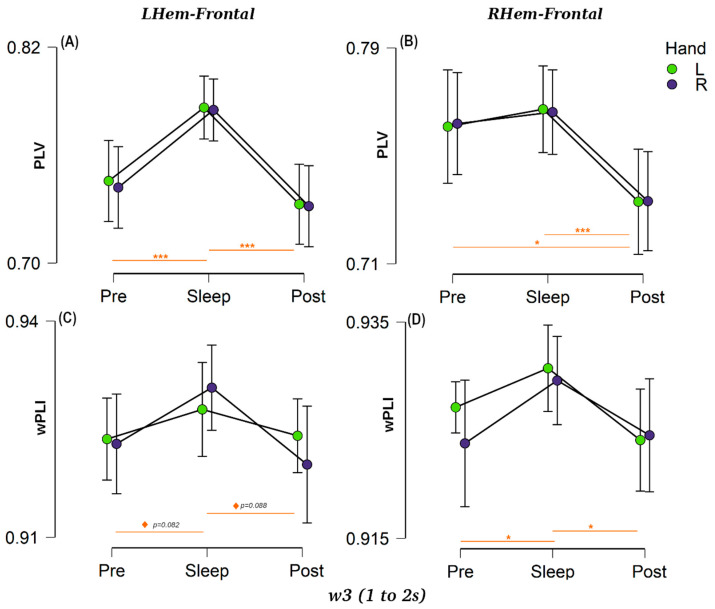
Hemisphere to frontal connectivity for *w*3 in each session (Pre-, Sleep and Post-sleep) for: (**A**) PLV left-hemisphere with frontal area (LHem–Frontal). (**B**) PLV right-hemisphere with frontal area (RHem–Frontal). (**C**) wPLI left-hemisphere with frontal area (LHem–Frontal). (**D**) wPLI right-hemisphere with frontal area (RHem–Frontal). Purple dots indicate the connectivity for R-hand trials and green dots for L-hand trials. Horizontal bars represent 95% confident intervals and sessions that are statistically significant are indicated by *** for *p* < 0.001, * for *p* < 0.05, and ^◊^ *p* < 0.10.

We calculated the connectivity values between the left hemisphere and the bilateral frontal area (LHem↔Frontal) as well as between the right hemisphere and the bilateral frontal area (RHem↔Frontal) for each window using both PLV and wPLI connectivity metrics (see Figure 3 for *w*3 and Supplementary Materials S1 for *w*1, *w*2, and full statistical results). An ANOVA analysis was performed for each session (3 levels) and each hand category (2 levels) as factors for each window and connectivity metric. The results showed a clear session effect, with a prominent increment in connectivity between the frontal and the lateral motor areas during our NREM sleep condition compared with the two wake sessions, thus confirming our predictions. This session effect was true for both hemispheres (RHem and LHem), particularly using the PLV measure (all *p* < 0.001, BF_10_ > 300), Figure 3A,B. The wPLI-based connectivity followed the same pattern (Figure 3C,D), but the effect was weaker (RHem: all *p* < 0.018, BF_10_ > 3.91, LHem: *p* < 0.047, BF_10_ > 1.85 except for time *w*2 where it was not significant). Various control analyses showed that these synchrony/connectivity results were not related to power differences (see Supplementary Materials S5).

A post-hoc analysis for the factor session showed clear differences between the Pre, Sleep and Post networks. Looking specifically at the right hemisphere (Figure 3B–D), PLV showed no significant differences between Pre and Sleep connectivity for RHem↔Frontal (all *p* > 0.52, BF_10_ > 0.210), but there was a large drop in Post connectivity compared with Pre for all three temporal windows (all *p* < 0.014, BF_10_ > 5.89). The same pattern was true between Sleep and Post (all temporal windows *p* < 0.001, BF_10_ > 157). On the other hand, using the wPLI measure (Figure 3D), RHem↔Frontal connectivity showed a positive difference between Pre and Sleep for *w*1 and *w*3 (*p* < 0.09, BF_10_ > 1.19) and a moderate difference for *w*2 (*p* = 0.013, BF_10_ = 7.35). Similar to PLV, there was also a strong difference between Sleep and Post sessions for the three windows of interest (all *p* < 0.028, BF_10_ > 4.70). Turning to the left hemisphere (Figure 3A–C), LHem↔Frontal PLV connectivity values showed a clear difference between wake and sleep sessions (all *p* < 0.001, BF_10_ > 240), (Figure 3A). The wPLI-based connectivity, despite following the same pattern as PLV for *w*1 and *w*3, with positive evidence for both Pre-Sleep (*w*1: *p* = 0.060, BF_10_ = 2.20, *w*3: *p* = 0.088, BF_10_ = 1.29) and Post-Sleep (*w*1: *p* = 0.082, BF_10_ = 2.20, *w*3: *p* = 0.082, BF_10_ = 1.89), did not achieve significance (Figure 3C). These differences between both connectivity metrics reflect that, although both metrics are measuring the same phase, they are sensitive to distinct sources of noise, as mentioned in the method section.

##### Between-Hemispheres (LHem—RHem) Connectivity

**Figure 4:**
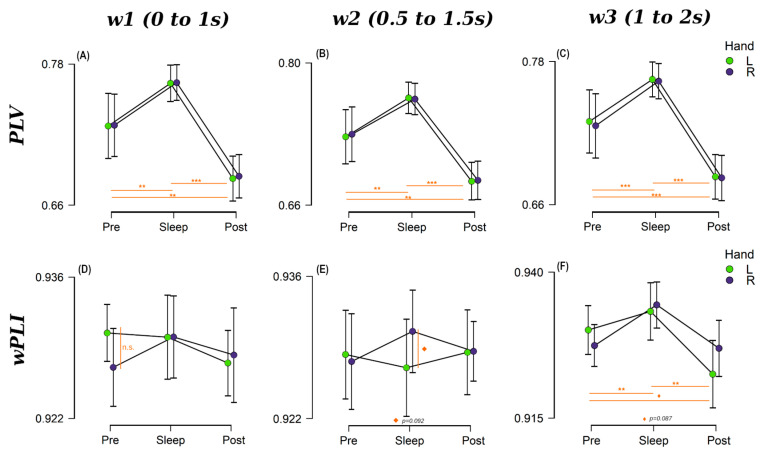
Between-hemisphere connectivity for each session (Pre-, Sleep and Post-sleep) at each 1s-long windows of interest (50% overlapping) for PLV (first row): (**A**) *w1*:0 to 1s from stimuli onset, (**B**) *w2* (0.5 s to 1.5 s) and (**C**) *w3* (1 s to 2 s) and wPLI (bottom row): (**D**) *w1*, (**E**) *w2* and (**F**) *w3*. Purple dots indicate the connectivity for R-handed trials and green dots for L-handed trials. Horizontal bars represent 95% confident intervals and those sessions statistically significant are indicated by *** for *p* < 0.001, ** for *p* < 0.01, ^◊^ *p* < 0.090, n.s. non-significant (*p* ≥ 0.10).

Next, we investigated the differences in connectivity between the two hemispheres for each of the three sessions, and for both R and L hands (Figure 4). Interestingly, across-hemisphere PLV (Figure 4A–C) showed an analogous behaviour to that seen in the above-mentioned analysis of hemisphere-frontal connectivity across the three windows of interest, with a large difference between Pre and Sleep (all *p* < 0.002, BF_10_ > 19.52) and between Sleep and Post (all *p* < 0.002, BF_10_ > 1.50E12). Additionally, there was a clear drop in brain connectivity during Post compared with Pre (all *p* < 0.001, BF_10_ > 41.45) for all the three windows (Figure 4, top row).

Using wPLI (Figure 4D–F), we found similar behaviour for *w*3 (Figure 4F), with moderate evidence for differences between Pre and Sleep (*p* = 0.014, BF_10_ = 5.96) and strong evidence for differences between Sleep and Post (*p* = 0.002, BF_10_ = 543.79), but the pattern of results differed for the other two time windows (0.199 < BF_10_ < 0.754) (Figure 4D,E). Whereas wPLI connectivity for the R-hand trials mirrored the PLV connectivity behaviour for all windows, this was not the case for L-hand trials. The latter presented a larger Pre-sleep connectivity in *w*1 (Figure 4D) and remained almost constant during the second window (Figure 4E). However, none of the differences between L and R hand connectivity values were statistically significant (lowest *p* = 0.092, BF_10_ = 0.94), as can be appreciated in Figure 4, bottom row. Refer to Supplementary Materials S2 for a full list of statistical results.

#### 3.2.2. Distinguishing Right- and Left-Handed Reactivations Using Connectivity

We were interested to know whether connectivity measures could be used to distinguish between different memories that are reactivated in sleep. Such reactivations were identified using a variety of methods such as machine learning [27,28], EEG-evoked potential amplitude similarity [46], and phase-related similarity between content-specific memory oscillations [25], and microstates [47] but not using brain connectivity metrics.

Our goal was therefore to determine whether the connectivity pattern occurring just after an R hand cue differed significantly from the connectivity pattern occurring just after a L hand cue in wake and sleep. We were also interested in how such hand-specific patterns might differ between sessions (Pre, Sleep and Post) and between windows (*w*1 to *w*3). Because this is a motor task, we expected connectivity to increase in the contralateral hemisphere with respect to the hand that was cued. Hence, for L hand trials, connectivity should be higher within right hemisphere than within left hemisphere, and the opposite should be true for R hand trials.

**Figure 5:**
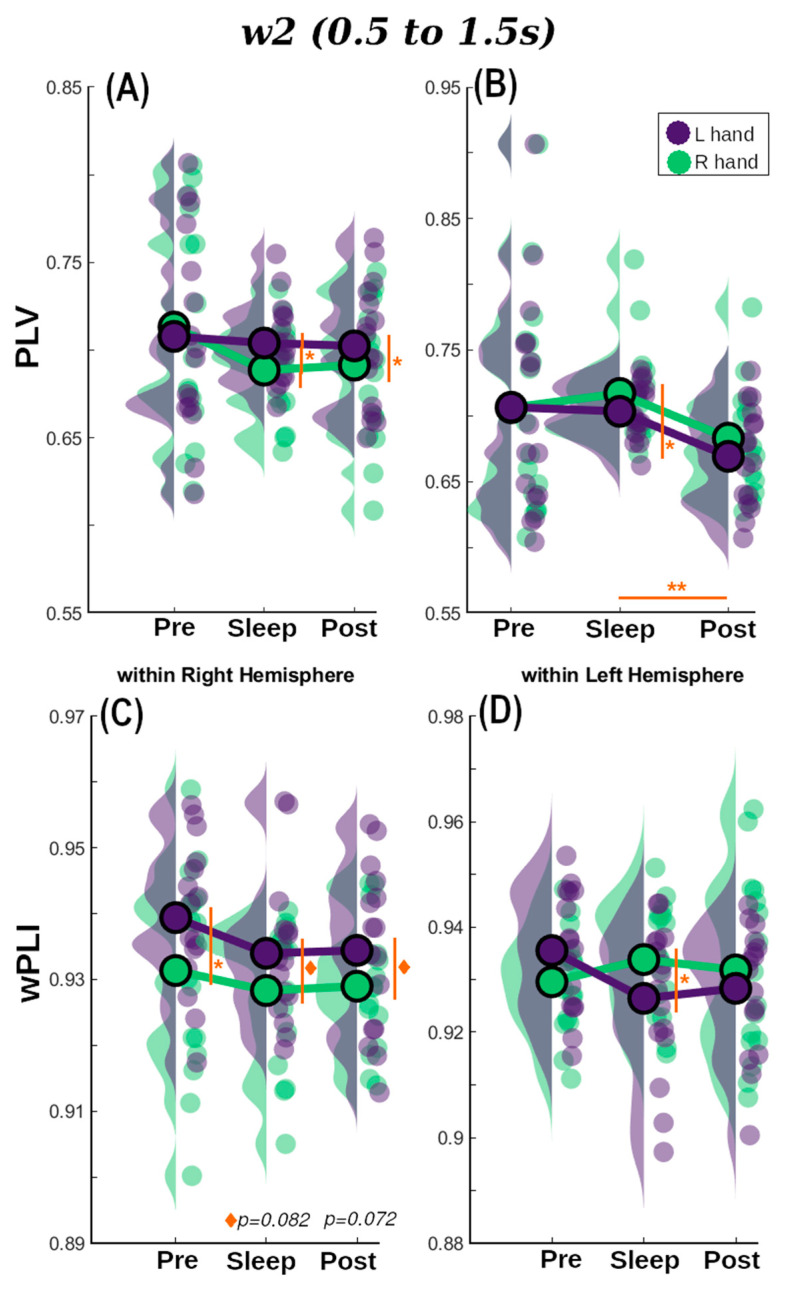
Repeated measures Anova results for *w*2 for: (**A**) PLV within right hemisphere, (**B**) PLV within left hemisphere, (**C**) wPLI within right hemisphere and (**D**) wPLI within left hemisphere. R hand condition is shown in green and L hand is represented in purple. Statistically significant results are highlighted in orange (* *p* < 0.05, ** *p* < 0.01, ^◊^ *p* < 0.09).

To examine this, we performed a similar analysis to that described in the previous sections, a RM-ANOVA (and its Bayesian version) with Session as repeated measure factor and Cued Hand (two levels: left and right) as a between-subjects factor. This was performed separately for measures of within-right and within-left hemisphere connectivity on each one of the three-time windows to gain statistical power. There were significant effects for Session, Cued Hand, and their interaction in different time windows and hemispheres (refer to Supplementary Materials S3 for the detailed statistical results). Finally, we performed a paired student *t*-test (Wilcoxon for non-normal distributions) comparing all the options to obtain a deeper understanding of the differences and once more, to gain statistical power, results are shown in Table S13 for within right hemisphere and Table S14 for within left hemisphere. Figure 5 shows the results for *w*2, refer to Supplementary Materials S3 for *w*1 and *w*3. 

For within right hemisphere PLV (Figure 5A for *w*2) there was no evidence for effects of Session, Cued Hand, or their interaction (0.109 < BF_10_ < 0.337). On the other hand, wPLI showed positive evidence for an effect of Session in *w*1 (*p* = 0.050, BF_10_ = 1.43), Cued Hand in *w* 2 (*p* = 0.039, BF_10_ = 1.60), Figure 5C, and the interaction between these in *w*3 (*p* = 0.002, BF_10_ = 2.67). For within-left hemisphere connectivity only PLV connectivity metric showed positive evidence for Session for the three windows of interest (BF_10_ = 1.62, 2.30, and 1.29, respectively). Post-hoc tests on PLV for Session resulted in strong evidence for differences in Sleep vs. Post for *w*1 (*p* = 0.009, BF_10_ = 9.69), that become larger for *w*2 (*p* = 0.002, BF_10_ = 40.59) (Figure 5B), and returned to moderate in *w*3 (*p* = 0.027, BF_10_ = 4.85).

PLV-based Pre-sleep connectivity (Figure 5A,B *w*2) was in general unable to differentiate between TMR cues for the R and L hands independently of the hemisphere or window analysed (lowest *p* = 0.12, 0.10 < BF_10_ < 0.38). Similar behaviour was found for *w*1 (BF_10_ = 0.199 and 0.435 for RHem and LHem, respectively) and *w*3 (BF_10_ = 0.087 and 0.11 for RHem and LHem, respectively) for wPLI-based connectivity values. However, there was moderate evidence suggesting a difference between hands for Pre *w*2 (*p* = 0.042, BF_10_ = 2.938) within the RHem.

In the Sleep session, both wPLI and PLV successfully detected significant differences between the TMR cues for R and L hands in both hemispheres, but this was not constant across windows. There is moderate evidence supporting differences between hands for the PLV-within RHem connectivity for *w*2 (*p* = 0.037, BF_10_ = 2.79) and *w*3 (*p* = 0.042, BF_10_ = 2.58). However, this turns into mere positive evidence for wPLI for the three windows (e.g., *w*2: *p* = 0.042, BF_10_ = 1.36). For the within-LHem measure, these differences are weaker for both connectivity metrics but still present. For PLV there is positive evidence for *w*1 (BF_10_ = 1.33) and *w*3 (BF_10_ = 1.09) and moderate for *w*2 (*p* = 0.005, BF_10_ = 2.38). Turning to the wPLI measure, only *w*2 and *w*3 showed positive evidence supporting the difference between hands (*p* = 0.042, BF_10_ = 1.91 and *p* = 0.049, BF_10_ = 1.70, respectively).

In contrast to the Pre-sleep connectivity, but similar to Sleep connectivity, Post connectivity values were also able to discern between TMR cues for the two hands in both hemispheres and both connectivity metrics. Moderate evidence was found for an effect of Cued Hand in PLV *w*2 (*p* = 0.022, BF_10_ = 3.75) for within-RHem, and *w*3 (*p* = 0.033, BF_10_ = 2.30) for within-LHem. Contrarily, there was moderate evidence for an effect of Cued Hand in wPLI within RHem for *w*3 (*p* = 0.012, BF_10_ = 5.11) but only weak evidence for *w*2 (*p* = 0.072, BF_10_ = 1.24). In LHem this behaviour was observed in *w*1 (*p* = 0.029, BF_10_ = 2.60) and *w*3 (*p* = 0.076, BF_10_ = 1.19).

In summary, both PLI and PLV were able to distinguish between L and R hands to a certain degree when used as within-hemisphere connectivity metrics. Results were particularly strong in *w*2 and *w*3, suggesting that reactivation may be occurring around the intersection of these two overlapping windows. This is 1.5 s after the stimulus onset and is therefore in line with [46] who found reactivation from 1.7 s from stimulus onset.

#### 3.2.3. Correlation with Behavioural Measures

Finally, we wanted to determine whether there was any relationship between behavioural measures (see Section 2) and the within-hemispheres connectivity values that allowed us to discriminate between TMR cues for the R and L hands. Prior work has found positive correlations between the extent of measurable reactivation and overnight consolidation see [25,46,48,49]. To search for such effects, we focused on Sleep and Post connectivity values since Pre (sleep) condition was not able to consistently differentiate between L and R hands. Based on the prior literature, combined with our own results, we expected correlations between behavioural improvements on the reactivated sequence (Re) and brain connectivity in *w*2 and *w*3 where the hand differences were strongest. All correlations were corrected using bootstrapping correction (*n* = 50,000).

**Figure 6:**
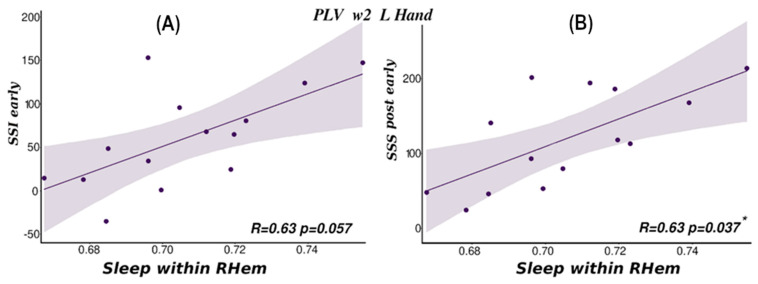
Correlation within right hemisphere (RHem–RHem) PLV connectivity values and behavioural results for reactivated sequences. (**A**) Overnight improvement (dependent on sequence specific improvement post early-SSI) and *w*2 connectivity for L hand. (**B**) Statistically significant correlation between post-sleep behaviour (sequence specific skill post early-SSS) and Sleep connectivity for *w*2 and L hand condition (* *p* < 0.05).

PLV-based RHem connectivity measures for L hand trials in *w*2 of the Sleep session correlated significantly with Post performance (sss_post_early, *Rho* = 0.63, *p* = 0.037), Figure 6A and showed a strong trend towards correlation with overnight performance improvement (ssi_early, *Rho* = 0.63, *p* = 0.057), Figure 6B on the reactivated sequence (see also Supplementary Materials S4). No other correlation was significant (all *p* > 0.061). These two findings are internally consistent since overnight improvement depends on post-sleep performance. This suggests that more connectivity in the motor area of the RHem during sleep predicts better performance on L hand trials the next morning. These results are also consistent with the behavioural results in which only the L hand condition showed an overnight behavioural improvement [26]. Furthermore, L hand movements should be associated with reactivation in the right hemisphere. On the other hand, there was no significant correlation with wPLI, supporting the idea that this measure taps into a slightly different form of connectivity.

Importantly, the above correlations were specific to *w*2. We found no correlations for either metric for *w*3 values (overall lowest *p* = 0.088). Additionally, and as expected from the connectivity analysis results, there were not significant correlations for *w*1 (overall lowest *p* = 0.118) for either connectivity metric. For the non-reactivated sequence, as expected, there were also no significant correlations.

## 4. Discussion

In this study, we showed that task-related neural connectivity between frontal and other cortical regions, as well as within each hemisphere, increases dramatically during slow wave sleep as compared to wake before or after sleep when examined directly after TMR stimulation. Connectivity also decreased from pre-to-post-sleep wake, supporting the idea of network-level consolidation in sleep. As our task involved motor imagery, we searched for hand-related responses, and found that within-hemisphere connectivity can be used to determine which hand was cued during sleep. This last finding suggests that our connectivity metrics could potentially be used as an index for measuring memory reactivation after a TMR cue during this deep stage of sleep.

### 4.1. Connectivity Differs Markedly between Pre-Sleep, Sleep, and Post-Sleep

Several recent studies have assessed sleep connectivity, particularly in the fields of consciousness and epilepsy, and all of them highlight that sleep is not globally uniform [1,2,3,4]. Thus, different cortical and subcortical regions are involved in different functions [50]. For instance, there is a consistent finding of top-down information flow from the parietal to the frontal regions during slow wave sleep [7,8]. Other work has shown an increment in frontal lobe connectivity during sleep [9]. Our observation of high connectivity between the frontal lobes and other cortical regions in SWS compared to wake sessions is broadly in line with these findings. Notably, this effect, which remained fairly constant for our three temporal windows (Figure 3 and Supplementary Materials S1), cannot be attributed to the specific memory content of the stimulation as it does not differ between L and R hands.

The same pattern was found for connectivity between motor areas, across hemispheres, with increased connectivity values for sleep compared with wake and no significant difference between hands (Figure 4). These results dovetail with previous studies finding a larger excitability within the motor network during sleep [9], and an increment in theta synchrony between central, temporal, and contralateral occipital homologues [1]. This pattern of results was particularly stable for PLV and wPLI in R hand trials. On the other hand, when we look at wPLI, L-hand connectivity appeared to be influenced by underlying processes that were not apparent for the R-hand trials, with more changes across sleep stages and windows. Previously, sleep-based connectivity studies have found right–left asymmetries among different brain regions. For instance, Betini et al. reported a flow of information from the left to the right hemisphere during sleep when compared with wake [51]. However, this cannot explain the differences between left- and right-handed trials in our dataset as it is only happening for one of the two hands; therefore, it cannot just be related to changes in the directionality of the connectivity during sleep. A more plausible explanation for this small difference is that left-handed movements produce activity in both hemispheres [52], which wPLI is able to capture, unlike PLV.

Another interesting behaviour found in the PLV measure is a consistent difference in between-hemisphere connectivity values for Pre and Post sessions. Overall connectivity is larger in Pre than Post, which could indicate that sleep and/or TMR somehow shapes the brain network, potentially optimising it for this particular task. A variety of neuroimaging results have demonstrated this type of network plasticity, presumably indicating that the network becomes more efficient as a result of consolidation [23,53,54,55]. In our data, this pattern is only significant in between-hemisphere connectivity and only using PLV. Connectivity between frontal channels and hemispheres has the same inverted-v pattern but only the right hemisphere–frontal values show significant pre-to-post-sleep differences. wPLI shows a numerically similar pattern but this is not significant, probably due to its larger sensitivity to the differences between hands.

### 4.2. Connectivity as a Measure of Replay

We demonstrated that connectivity metrics based on phase locking in the brain (PLV and wPLI) can consistently differentiate between motor imagery relating to finger presses using the right and left hands. However, that difference between the hands is only apparent when using connectivity values calculated within each hemisphere, and not for any other region analysed. This is in line with the existing literature since motor imagery is hemisphere-dependent [56]. Nevertheless, these differences only appear consistently during sleep and post-sleep sessions, suggesting sleep is necessary to consolidate the differences between hands.

We also found that the temporal window from stimulus onset to 1 s provides a less precise differentiation between hands than later windows. Previous TMR literature investigating the time course of the reactivation effect found similar patterns. Cairney et al, found significant differences between 1.76 s and 2 s of stimuli onset [46]. Others discovered a recurrent pattern with two distinct periods, one early and one late, from stimuli onset [25]. Zhang et al., also discovered two clusters, but only the later one (ranging from 0.5 to 1.2 s) was related to behavioural performance [57]. In our case, the two overlapping windows ranging from 0.5 to 1.5 s and from 1 to 2 s allow us to infer that reactivation is occurring during the overlapping time of the window, hence between 1 and 1.5 s. However, only connectivity during the second window showed significant relationships with behavioural performance.

All these findings support the idea that sleep is not homogenous and has very specialised functions. We can conclude that TMR only affected the brain connectivity of regions involved in the task. Meaning that, given this was a motor imagery task, the within-hemisphere connectivity behaved differently when we cued L and R hands, but the rest of the regions we analysed showed the same patterns of connectivity as a normal wake–sleep study that did not involve TMR cueing, with no differences between the conditions. However, TMR also seems to have a less clear effect on next morning connectivity when compared with the pre-sleep network.

### 4.3. Caveats and Limitations

We would like to point out that this work is limited by both the number of EEG channels which were recorded and the small number of participants. However, the results are broadline in line with the previous literature and the novel connectivity findings are strongly correlated with the behavioural measures, we feel it is reasonable to take the data at face value while bearing these limitations in mind.

Another issue relates to the use of PLV connectivity on the scalp electrodes as this measure is vulnerable to contamination by the effects of volume conduction. To mediate this, we used a second phase-based metric, wPLI, that is less prone to such contamination. In general, the two connectivity metrics show similar results. Furthermore, we always compared between the two conditions (L versus R hand) or between the sessions (pre–sleep–post), which should all be equally affected by volume conduction, so these issues should have dropped out in the comparison and should therefore not interfere with our results.

## 5. Conclusions

In this study, we showed that task related neural connectivity increases dramatically during slow wave sleep as compared to wake before or after sleep when examined directly after TMR stimulation. Connectivity also decreased from pre-to post- sleep wake, supporting the idea of network-level consolidation in sleep. As our task involved motor imagery, we searched for hand-related responses, and found that within-hemisphere connectivity can be used to determine which hand was cued during sleep. This finding means that brain connectivity can be used a possible marker of how successful the memory reactivation was.

## Data Availability

The raw data can be found at 10.5281/zenodo.10513358.

## Supplementary Materials

The following supporting information can be downloaded at: https://www.mdpi.com/article/10.3390/brainsci14020114/s1, Supplementary Materials S1: Hemispheres-Frontal connectivity, Supplementary Materials S2: Between hemispheres connectivity, Supplementary Materials S3: Within hemispheres connectivity, Supplementary Materials S4: Behavior-Connectivity correlations, Supplementary Materials S5: Power analysis.
