# Supplementary material for: Effects of Targeted Memory Reactivation on Cortical Networks
Source: Brain Sci. 2024 Jan 23;14(2):114. doi: 10.3390/brainsci14020114 (PMC10886727; doi:10.3390/brainsci14020114)
Supplement: Supplementary file 1 [file brainsci-14-00114-s001.zip › brainsci-2802015-supplementary.pdf]

brain sciences

Supplementary Materials S1: Hemispheres- Frontal connectivity

Table S1 Repeated measures Anova with Session as repeated factors and Hand as between factors for the three windows of interest (w1, w2 and w3) and the two-connectivity metrics, PLV (left) and wPLI (right) for the right hemisphere-frontal connectivity. Statistically significant values are shadowed in grey with the respective p values in bold.

RHem - Frontal	PLV			wPLI		
	w1	***w2***	***w3***	***w1***	***w2***	***w3***
Session	F=8.8,**p<0.001**<br>BF_{10}=**697.17,**<br>BF_{incl}=482.10	F=8.2,**p<0.001**<br>BF_{10}=**304.78,**<br>BF_{incl}=482.10	F=8.2,**p<0.001**<br>BF_{10}=**637.80,**<br>BF_{incl}=439.63	F=4.49,**p=0.015**<br>BF_{10}=**6.020,**<br>BF_{incl}=4.13	F=5.29,**p=0.008**<br>BF_{10}=**11.562,**<br>BF_{incl}=8.136	F=4.30,**p=0.018**<br>BF_{10}=**3.910,**<br>BF_{incl}=2.755
Hand	F=0.02,p=0.87<br>BF_{10}=0.248,<br>BF_{incl}=0.196	F=0.02,p=0.97<br>BF_{10}=0.279,<br>BF_{incl}=0.198	F=0.00, p=0.99<br>BF_{10}=0.249<br>BF_{incl}=0.194	F=0.05,p=0.816<br>BF_{10}=0.277,<br>BF_{incl}=0.206	F=0.31,p=0.584<br>BF_{10}=0.281,<br>BF_{incl}=0.231	F=0.402,p=0.67<br>BF_{10}=0.317,<br>BF_{incl}=0.251
Session<br>*Hand	F=0.04,p=0.96<br>BF_{10}=177.70,<br>BF_{incl}=0.127	F=0.02,p=0.98<br>BF_{10}=78.56,<br>BF_{incl}=0.128	F=0.01, p=0.992<br>BF_{10}=160.69<br>BF_{incl}=0.127	F=0.14,p=0.868<br>BF_{10}=1.612,<br>BF_{incl}=0.127	F=0.40,p=0.671<br>BF_{10}=3.340,<br>BF_{incl}=0.181	F=4.02,p=0.529<br>BF_{10}=1.246,<br>BF_{incl}=0.178

Table S2 Repeated measures Anova with Session as repeated factors and Hand as between factors for the three windows of interest (w1, w2 and w3) and the two-connectivity metrics, PLV (left) and wPLI (right) for the right hemisphere-frontal connectivity. Statistically significant values are shadowed in grey with the respective p values in bold.

LHem-<br>Frontal	PLV			wPLI		
	w1	***w2***	***w3***	***w1***	***w2***	***w3***
Session	F=16.8,**p<0.001**<br>BF_{10}=**285202.44,**<br>BF_{incl}=197355.38	F=15.1,**p<0.001**<br>**BF_{10}=66300.06**<br>BF_{incl}=45682.4	F=16.5,**p<0.001**<br>**BF_{10}=188967.1**<br>BF_{incl}=131181.0	F=3.38,**p=0.040**<br>BF_{10}=**1.85,**<br>BF_{incl}=1.13	F=2.43,**p=0.096**<br>BF_{10}=0.868<br>BF_{incl}=0.605	F=3.21,**p=0.047**<br>BF_{10}=**2.12,**<br>BF_{incl}=1.486
Hand	F=0.001,p=0.97<br>BF_{10}=0.250,<br>BF_{incl}=0.198	F=0.016,p=0.90<br>BF_{10}=0.256, BF_{incl}=0.199	F=0.06,p=0.79<br>BF_{10}=0.252 BF_{incl}=0.203	F=0.05,p=0.825<br>BF_{10}=0.296<br>BF_{incl}=0.243	F=0.00,p=0.930<br>BF_{10}=0.279<br>BF_{incl}=0.204	F=0.06,p=0.803<br>BF_{10}=0.262<br>BF_{incl}=0.208
Ses-<br>sion*Hand	F=0.010,p=0.99<br>BF_{10}=73413.86,<br>BF_{incl}=0.127	F=0.013,p=0.99<br>BF_{10}=16894.79,<br>BF_{incl}=0.137	F=0.01,p=0.99<br>BF_{10}=49308.56<br>BF_{incl}=0.137	F=1.09,p=0.342<br>BF_{10}=0.468<br>BF_{incl}=0.195	F=0.42,p=0.658<br>BF_{10}=0.240<br>BF_{incl}=0.087	F=0.67,p=0.514<br>BF_{10}=0.551<br>BF_{incl}=0.164

Table S3 Post-hoc analysis for Session from the right hand-frontal connections. Statistically significant values are shadowed in grey with the respective p values in bold and p-values less than 0.10 are only highlighted in bold. C=Cohens'd value.

RHem -<br>Frontal	PLV			wPLI		
	w1	***w2***	***w3***	***w1***	***w2***	***w3***
Pre-Sleep	t=-0.48, p=0.64<br>C=-0.08<br>BF_{10}=0.21	t=-0.52, p=0.621<br>C=-0.09<br>BF_{10}=0.214	t=-0.65, p=0.52<br>C=-0.12<br>BF_{10}=0.230	t=-2.05, **p=0.09**<br>C=-0.36<br>BF_{10}=**1.193**	t=-2.98, **p=0.013**<br>C= -0.58<br>BF_{10}=**7.35**	t=-2.54, **p=0.032**<br>C= -0.45<br>BF_{10}=**2.954**
Pre-Post	t=3.07, **p=0.009**<br>C=0.543	t=2.88, **p=0.014**<br>C=0.51	t=2.98, **p=0.011**<br>C=0.53	t=0.41, p=0.68<br>C=0.07	t=-0.16, p=0.87<br>C= -0.03	t=0.49, p=0.62<br>C=0.09

	BF_{10}=8.887	BF_{10}=5.890	BF_{10}=7.35	BF_{10}=0.204	BF_{10}=0.191	BF_{10}=0.212
Sleep-Post	t=4.43, **p<0.001**<br>C=0.78<br>**BF_{10}=236.56**	t=4.27, **p<0.001**<br>C=0.75<br>**BF_{10}=157.95**	t=4.40, **p<0.001**<br>C=0.78<br>**BF_{10}=220.77**	t=3.53, **p=0.04**<br>C=0.62<br>**BF_{10}=25.27**	t=3.09, **p=0.013**<br>C=0.546<br>**BF_{10}=9.27**	t=2.77, **p=0.028**<br>C=0.49<br>BF_{10}=**4.701**

Table S4 Post-hoc analysis for Session from the left hand-frontal connections. Statistically significant values are shadowed in grey with the respective p values in bold. p-values less than 0.10 are highlighted in bold.

LHem Frontal	**PLV**			**wPLI**		
	w1	***w2***	***w3***	***w1***	***w2***	***w3***
Pre-Sleep	t=-4.44,**p<0.001**<br>C= -0.78<br>**BF_{10}=240.94**	t=-4.44,**p<0.001**<br>C= -0.78<br>**BF_{10}=242.97**	t=-4.69,**p<0.001**<br>C= -0.83<br>**BF_{10}=471.61**	t=-2.39,**p=0.069**<br>C= -0.43<br>**BF_{10}=2.202**	t=-1.82,p=0.234<br>C=-0.31<br>BF_{10}=0.824	t=-2.10,**p=0.088**<br>C=-0.37<br>BF_{10}=**1.294**
Pre-Post	t=1.22, p=0.232<br>C=0.21<br>BF_{10}=0.371	t=1.02, p=0.317<br>C=0.18<br>BF_{10}=0.303	t=1.06, p=0.299<br>C=0.18<br>BF_{10}=0.315	t=0.16, p=0.869<br>C=0.029<br>BF_{10}=0.191	t=-1.78, p=0.234<br>C=-0.31<br>BF_{10}=0.772	t=0.39, p=0.699<br>C=0.029<br>BF_{10}=0.203
Sleep-Post	t=6.23, **p<0.001**<br>C =1.10<br>**BF_{10}=26781.00**	t=5.99, **p<0.001**<br>C=1.05<br>**BF_{10}=13344.29**	t=6.08, **p<0.001**<br>C=1.07<br>**BF_{10}=17817.57**	t=2.13, **p=0.082**<br>C=0.37<br>BF_{10}=**1.363**	t=0.08, p=0.939<br>C=0.01<br>BF_{10}=0.189	t=2.31, **p=0.082**<br>C=0.41<br>BF_{10}=**1.899**

Table S5 Paired t-test for Hand (Right versus Left) for PLV (top row) and wPLI (bottom row) connectivity metrics. Statistically significant values are shadowed in grey with the respective p values in bold. p-values less than 0.10 are highlighted in bold.

LHem - F	PLV				
	Statistic	df	*p*	Effect size	BF10
Pre w1	-0.436	15	0.669	-0.109	0.27
Sleep w1	0.013	15	0.990	0.003	0.25
Post w1	0.974	15	0.345	0.244	0.38
Pre w2	-1.200	15	0.249	-0.300	0.47
Sleep w2	-0.316	15	0.756	-0.079	0.26
Post w2	0.275	15	0.787	0.069	0.26
Pre w3	-1.43	15	0.174	-0.357	0.600
Sleep w3	-0.775	15	0.450	-0.194	0.332
Post w3	-0.617	15	0.547	-0.154	0.302

RHem - F	PLV				
	Statistic	df	*p*	Effect size	BF10
Pre w1	1.248	15	0.231	0.312	0.49
Sleep w1	-1.681	15	0.113	-0.420	0.81
Post w1	1.772	15	**0.097**	0.443	0.91
Pre w2	82.00	15	0.495	0.206	0.48
Sleep w2	-0.784	15	0.445	-0.206	0.33
Post w2	-0.049	15	0.961	-0.012	0.25
Pre w3	0.689	15	0.501	0.172	0.315
Sleep w3	-1.054	15	0.309	-0.264	0.412
Post w3	0.121	15	0.906	0.030	0.257

LHem - F	wPLI				
	Statistic	df	*p*	Effect size	BF10
Pre w1	0.253	15	0.804	0.063	0.486
Sleep w1	-0.295	15	0.772	-0.074	0.257
Post w1	0.530	15	0.604	0.133	0.258
Pre w2	0.964	15	0.350	0.241	0.290
Sleep w2	-0.250	15	0.806	-0.062	0.330
Post w2	0.219	15	0.830	0.055	0.363
Pre w3	-1.326	15	0.205	-0.331	0.314
Sleep w3	-0.577	15	0.573	-0.144	0.390
Post w3	0.168	15	0.869	0.042	0.398

RHem - F	wPLI				
	Statistic	df	*p*	Effect size	BF10
Pre w1	-1.229	15	0.238	-0.307	0.263
Sleep w1	-0.538	15	0.598	-0.135	0.382
Post w1	0.686	15	0.503	0.172	0.536
Pre w2	0.103	15	0.919	0.026	0.264
Sleep w2	0.764	15	0.457	0.191	0.263
Post w2	-0.991	15	0.337	0.191	0.296
Pre w3	-0.149	15	0.869	0.042	0.289
Sleep w3	0.899	15	0.383	0.225	0.261
Post w3	-1.015	15	0.326	-0.254	0.259

Wilcoxon signed-rank test is used when normality (Shapiro-Wilk test) assumption is violated. In this case effect size is given by the matched rank bi-serial correlation. Otherwise, Student t-test and Cohen’s d for measure effect size are used.

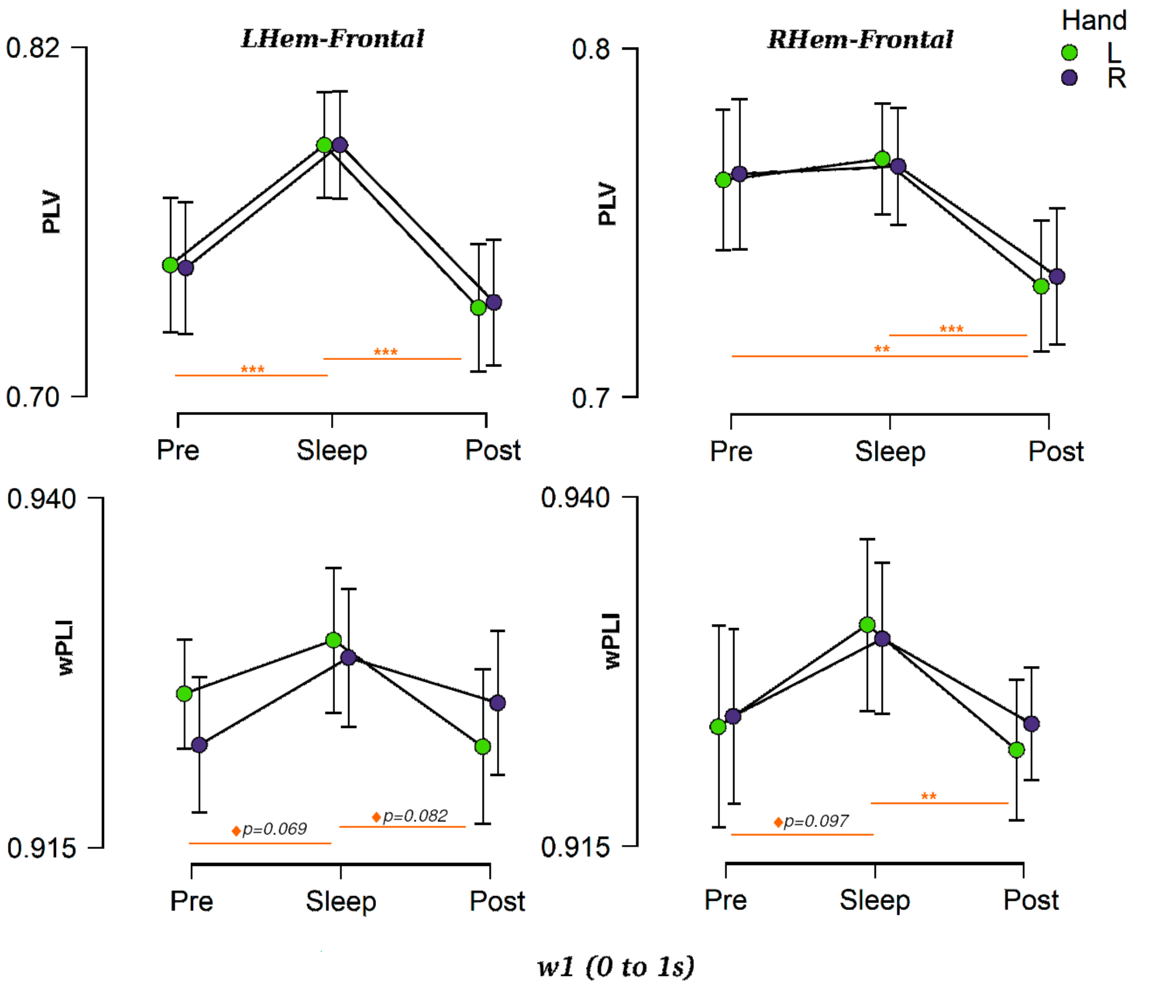

*Figure S1. Hemisphere to frontal connectivity for w1 in each session (Pre, Sleep and Post sleep) for PLV (up) and wPLI (down), left hemisphere-frontal (LHem-F) on the first column and right hemisphere - frontal (RHem-F) in the second column. Purple indicates the connectivity for R hand trials and green dots for L hand trials. Horizontal bars represent 95% confident intervals and those sessions statistically significant are indicated by *** for p<0.001, ** p<0.01, * for p<0.05 and ◊ p<0.10.*

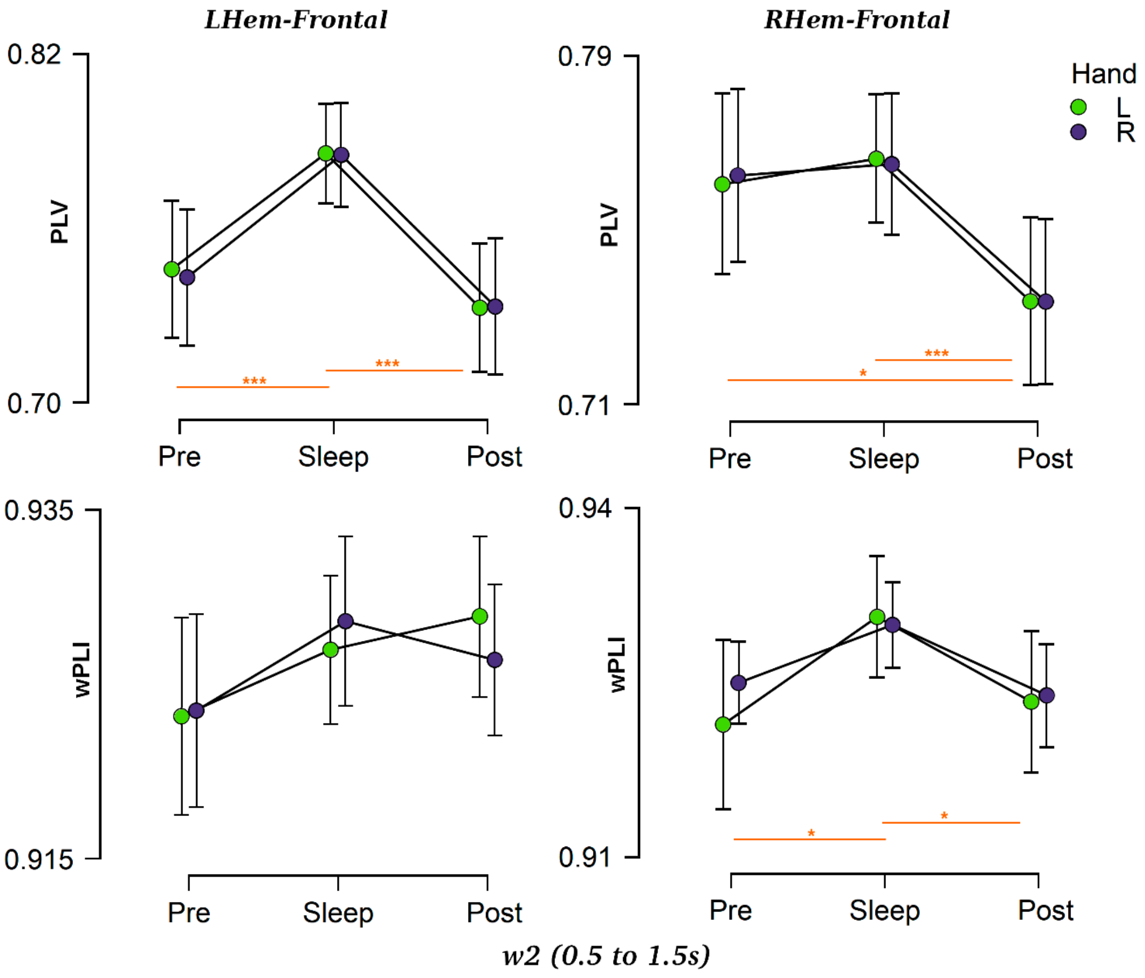

*Figure S2. Hemisphere to frontal connectivity for w2 in each session (Pre, Sleep and Post sleep) for PLV (up) and wPLI (down), left hemisphere -frontal (LHem-F) on the first column and right hemisphere -frontal (RHem-F) in the second column. Purple dots indicate the connectivity for R hand trials and green dots for L hand trials. Horizontal bars represent 95% confident intervals and those sessions statistically significant are indicated by *** for p<0.001, ** p<0.01, * for p<0.05 and ◊ p<0.10.*

Supplementary Materials S2: between hemispheres connectivity

Table S6 RM-Anova results for between hemispheres connectivity (RHem-LHem), PLV (left) and wPLI (right) for each window of interest (w1 to w3).

RHem - LHem	PLV			wPLI		
	w1	***w2***	***w3***	***w1***	***w2***	***w3***
Session	F=33.0, **p<0.001** **BF_{10}=4.33E10** BF_{incl}=2.98E10	F=34.38,**p<0.001** **BF_{10}=1.15E11** BF_{incl}=8.31E10	F=35.3,**p<0.001** **BF_{10}=1.87E11** BF_{incl}=1.30E11	F=0.73,p=0.48 BF_{10}=0.180 BF_{incl}=0.128	F=0.09,p=0.91 BF_{10}=0.105 BF_{incl}=0.074	F=8.92, **p<0.001** **BF_{10}=186.766** BF_{incl}=137.87
Hand	F=0.02, p=0.88 BF_{10}=0.257 BF_{incl}=0.199	F=0.01, p=9.06 BF_{10}=0.258 BF_{incl}=0.243	F=0.08, p=0.77 BF_{10}=0.253 BF_{incl}=0.207	F=0.25,p=0.62 BF_{10}=0.295 BF_{incl}=0.206	F=0.32,p=0.58 BF_{10}=0.297 BF_{incl}=0.202	F=0.24,p=0.625 BF_{10}=0.283 BF_{incl}=0.280

Session*Hand	F=0.00, p=0.98<br>BF_{10}=1.11E10<br>BF_{incl}=0.130	F=0.002, p=0.98<br>BF_{10}=3.78E10<br>BF_{incl}=0.105	F=0.01, p=0.98<br>BF_{10}=05.40E10<br>BF_{incl}=0.128	F=0.76,p=0.47<br>BF_{10}=0.054<br>BF_{incl}=0.041	F=0.66,p=0.52<br>BF_{10}=0.031<br>BF_{incl}=0.023	F=1.35,p=0.265<br>BF_{10}=53.262<br>BF_{incl}=0.418

Table S7. Post-hoc analysis for Session. Statistically significant values are shadowed in grey with the respective p values in bold and p-values less than 0.10 are highlighted in bold.

RHem - LHem	PLV			wPLI		
	w1	***w2***	***w3***	***w1***	***w2***	***w3***
Pre-Sleep	t=-3.42,**p=0.002**<br>C=-0.60<br>**BF_{10}=19.52**	t=-3.46, **p=0.002**<br>C=-0.61<br>**BF_{10}=21.37**	t=-3.51,**p=0.001**<br>C=-0.62<br>**BF_{10}=24.72**	t=-0.75,p=0.92<br>C= -0.13<br>BF_{10}=0.245	t=-0.37, p=1<br>C= -0.13<br>BF_{10}=0.201	t=-2.89, **p=0.014**<br>C= -0.51<br>**BF10=5.965**
Pre-Post	t=3.73,**p=0.002**<br>C=0.66<br>**BF_{10}=41.45**	t=3.84, **p=0.001**<br>C=0.67<br>**BF_{10}=54.21**	t=3.85,**p=0.001**<br>C=0.68<br>**BF_{10}=55.03**	t=0.52,p=0.92<br>C= -0.09<br>BF_{10}=0.214	t=-0.33, p=1<br>C= -0.06<br>BF_{10}=0.199	t=1.76, **p=0.087**<br>C= 0.31<br>BF_{10}=0.754
Sleep-Post	t=14.0,**p<0.001**<br>C=2.48<br>**BF_{10}=1.19E12**	t=14.1, **p<0.001**<br>C=-2.50<br>**BF_{10}=1.50E12**	t=14.9,**p<0.001**<br>C=-2.66<br>**BF_{10}=5.4E12**	t=1.10, p=0.841<br>C=0.19<br>BF_{10}=0.328	t=1.12, p=1<br>C= 0.22<br>BF_{10}=0.190	t=3.76, **p=0.002**<br>C= 0.66<br>**BF10=543.793**

Table S8 Paired t-test for Hand (Right versus Left) in wPLI connectivity. Statistically significant values are shadowed in grey with the respective p values in bold.

**RHem - LHem**	**wPLI**				
	**Statistic**	**df**	***p***	**Effect size**	**BF_{10}**
Pre w1	-1.754	15	0.100	-0.439	0.891
Sleep w1	0.010	15	0.992	0.003	0.255
Post w1	0.331	15	0.745	0.083	0.268
Pre w2	-0.319	15	0.754	-0.080	0.267
Sleep w2	1.801	15	0.092	0.450	0.947
Post w2	63.00	15	0.821	-0.074	0.256
Pre w3	-0.872	15	0.397	-0.218	0.356
Sleep w3	0.394	15	0.699	0.099	0.274
Post w3	1.299	15	0.214	0.325	0.521

Supplementary Materials S3: within hemispheres connectivity

Table S9 RM-Anova results for within right hemisphere (RHem) connectivity, PLV (left) and wPLI (right) for each window of interest (w1 to w3).

within RHem	**PLV**			**wPLI**		
	w1	***w2***	***w3***	***w1***	***w2***	***w3***
Session	F=0.46 ,p=0.63<br>BF_{10}=0.198<br>BF_{incl}=0.137	F=1.37, p=0.26<br>BF_{10}=0.302<br>BF_{incl}=0.214	F=0.18,p=0.84<br>BF_{10}=0.109<br>BF_{incl}=0.076	F=3.15, **p=0.05**<br>BF_{10}=**1.427**<br>BF_{incl}=1.044	F=1.49, p=0.23<br>BF_{10}=0.338<br>BF_{incl}=0.249	F=0.47,p=0.62<br>BF_{10}=0.136<br>BF_{incl}=0.919

Hand	F=0.002,p=0.96<br>BF_{10}=0.282<br>BF_{incl}=0.193	F=0.46, p=0.50<br>BF_{10}=0.337<br>BF_{incl}=0.238	F=0.25,p=0.62<br>BF_{10}=0.328<br>BF_{incl}=0.223	F=0.96, p=0.33<br>BF_{10}=0.385<br>BF_{incl}=0.310	F=4.68, **p=0.039**<br>BF_{10}=**1.600**<br>BF_{incl}=1.112	F=0.659,p=0.42<br>BF_{10}=0.340<br>BF_{incl}=1.205
Session*Hand	F=0.034,p=0.96<br>BF_{10}=0.056<br>BF_{incl}=0.023	F=0.64, p=0.53<br>BF_{10}=0.102<br>BF_{incl}=0.60	F=0.25,p=0.77<br>BF_{10}=0.036<br>BF_{incl}=0.019	F=0.92, p=0.40<br>BF_{10}=0.564<br>BF_{incl}=0.211	F=0.15, p=0.86<br>BF_{10}=0.537<br>BF_{incl}=0.109	F=6.90,**p=0.002**<br>BF_{10}=**2.667**<br>BF_{incl}=4.384

Table S10 RM-Anova results for within left hemisphere (LHem) connectivity, PLV (left) and wPLI (right) for each window of interest (w1 to w3).

within LHem	**PLV**			**wPLI**		
	w1	***w2***	***w3***	***w1***	***w2***	***w3***
Session	F=3.26 **,p=0.057**<br>BF_{10}=**1.623**<br>BF_{incl}=1.18	F=3.43**, p=0.039**<br>BF_{10}=**2.306**<br>BF_{incl}=1.60	F=2.73,**p=0.073**<br>BF_{10}=**1.292**<br>BF_{incl}=0.896	F=0.22, p=0.80<br>BF_{10}=0.136<br>BF_{incl}=0.094	F=0.35, p=0.71<br>BF_{10}=0.131<br>BF_{incl}=0.126	F=0.34,p=0.71<br>BF_{10}=0.128<br>BF_{incl}=0.134
Hand	F=0.001,p=0.97<br>BF_{10}=0.260<br>BF_{incl}=0.191	F=0.57, p=0.45<br>BF_{10}=0.313<br>BF_{incl}=0.233	F=0.21, p=0.64<br>BF_{10}=0.272<br>BF_{incl}=0.201	F=4.27, **p=0.047**<br>BF_{10}=0.852<br>BF_{incl}=0.574	F=0.49, p=0.49<br>BF_{10}=0.295<br>BF_{incl}=0.241	F=0.31,p=0.58<br>BF_{10}=0.293<br>BF_{incl}=0.251
Session*Hand	F=0.012,p=0.97<br>BF_{10}=0.417<br>BF_{incl}=0.088	F=0.16, p=0.85<br>BF_{10}=0.710<br>BF_{incl}=0.120	F=0.05, p=0.95<br>BF_{10}=0.358<br>BF_{incl}=0.081	F=0.56, p=0.58<br>BF_{10}=0.101<br>BF_{incl}=0.047	F=2.06, p=0.14<br>BF_{10}=0.038<br>BF_{incl}=0.206	F=3.38,**p=0.041**<br>BF_{10}=0.096<br>BF_{incl}=0.262

Table S11. Post-hoc analysis for Session (Pre, Sleep, Post) for within right hemisphere (RHem). Statistically significant values are shadowed in grey with the respective p values in bold, p-values less than 0.10 are highlighted in bold. C=Cohen's d for measures of effect size.

within RHem	**PLV**			**wPLI**		
	w1	***w2***	***w3***	***w1***	***w2***	***w3***
Pre –Sleep	t=-1.05, p=1<br>C=-0.01<br>BF_{10}=0.361	t=1.34, p=0.57<br>C=0.24<br>BF_{10}=0.427	t=0.53, p=1<br>C=0.09<br>BF_{10}=0.216	t=2.92, p=0.10<br>C=0.39<br>BF_{10}=1.518	t=1.54, p=0.40<br>C=0.27<br>BF_{10}=0.545	t=0.67, p=1<br>C=0.11<br>BF_{10}=0.229
Pre-Post	t=0.92, p=1<br>C=0.16<br>BF_{10}=0.275	t=1.34, p=0.57<br>C=0.24<br>BF_{10}=0.427	t=0.51, p=1<br>C=0.07<br>BF_{10}=0.205	t=0.85, p=0.40<br>C=0.15<br>BF_{10}=0.263	t=1.28, p=0.42<br>C=0.22<br>BF_{10}=0.400	t=0.74, p=1<br>C=0.13<br>BF_{10}=0.244
Sleep-Post	t=0.87, p=1,<br>C=0.15<br>BF_{10}=0.203	t=-0.08, p=0.94<br>C=-0.01<br>BF_{10}=0.189	t=-0.15, p=1<br>C=-0.03<br>BF_{10}=0.191	t=-1.85, p=0.15<br>C=-0.33<br>BF_{10}=0.826	t=-0.25, p=0.80<br>C=-0.04<br>BF_{10}=0.194	t=0.24, p=1<br>C=0.04<br>BF_{10}=0.194

Table S12. Post-hoc analysis for Session (Pre, Sleep, Post) for within left hemisphere (LHem). Statistically significant values are shadowed in grey with the respective p values in bold, p-values less than 0.10 are highlighted in bold. C=Cohen's d for measures of effect size.

within LHem	**PLV**	**wPLI**

	w1	***w2***	***w3***	***w1***	***w2***	***w3***
Pre –Sleep	t=-1.09, p=0.91<br>C=-0.019<br>BF_{10}=0.192	t=-0.31, p=0.82<br>C=0.04<br>BF_{10}=0.194	t=-0.04, p=0.97<br>C=-0.01<br>BF_{10}=0.189	t=-0.23, p=1<br>C=-0.04<br>BF_{10}=0.194	t=0.71, p=1<br>C=0.12<br>BF_{10}=0.238	t=-0.57, p=1<br>C=-0.10<br>BF_{10}=0.220
Pres-Post	t=2.04, p=0.099<br>C=0.361<br>BF_{10}=0.884	t=1.97, p=0.12<br>C=0.35<br>BF_{10}=1.040	t=0.33, p=0.15<br>C=0.33<br>BF_{10}=0.853	t=-0.61, p=1<br>C=-0.11<br>BF_{10}=0.225	t=0.73, p=1<br>C=0.13<br>BF_{10}=0.241	t=-0.75, p=1<br>C=-0.13<br>BF_{10}=0.244
Sleep-Post	t=3.20, **p=0.009**<br>C=0.567<br>**BF_{10}=9.687**	t=3.72, **p=0.002**<br>C=0.66<br>**BF_{10}=40.595**	t=2.79, **p=0.027**<br>C=0.493<br>**BF_{10}=4.853**	t=-0.42, p=1<br>C=-0.07<br>BF_{10}=0.205	t=0.01, p=1<br>C=0.02<br>BF_{10}=0.189	t=-0.20, p=1<br>C=-0.03<br>BF_{10}=0.192

Table S13. Statistical results from paired t-test comparing R vs. L hands for each window and session for within right hemisphere connectivity. Wilcoxon signed-rank test is used when normality (Shapiro-Wilk test) assumption is violated. In this case, effect size is given by the matched rank bi-serial correlation. Otherwise, paired student t-test and Cohen's d values are stated. All tests were one-tailed.

**R vs. L hand**		**within RHem**							
		**PLV**				**wPLI**			
		**Statistic**	***p***	**Effect size**	**BF_{10}**	**Statistic**	***p***	**Effect size**	**BF_{10}**
Pre	w1	-1.18	0.128	-0.29	0.12	0.365	0.640	0.091	0.199
	w2	1.86	0.959	0.46	0.10	-1.85	**0.042**	-0.46	**2.938**
	w3	95.0	0.920	0.39	0.21	2.65	0.991	0.66	0.087
Sleep	w1	0.80	0.784	0.20	0.36	-1.603	**0.042**	-0.401	**1.360**
	w2	33.0	**0.037**	-0.51	**2.79**	-1.46	**0.082**	-0.36	**1.130**
	w3	34.0	**0.042**	-0.50	**2.58**	-1.55	**0.070**	-0.38	**1.276**
Post	w1	-0.60	0.277	-0.15	0.51	-0.817	0.213	-0.20	0.529
	w2	29.0	**0.022**	-0.57	**3.75**	-1.53	**0.072**	-0.38	**1.247**
	w3	61.0	0.372	-0.10	0.53	-2.49	**0.012**	-0.62	**5.115**

Table S14. Statistical results from paired t-test comparing R vs. L hands for each window and session for within left hemisphere connectivity. Wilcoxon signed-rank test is used when normality (Shapiro-Wilk test) assumption is violated. In this case, effect size is given by the matched rank bi-serial correlation. Otherwise, paired student t-test and Cohen's d values are stated. All tests were one-tailed.

**R vs. L hand**		**within LHem**							
		**PLV**				**wPLI**			
		**Statistic**	***p***	**Effect size**	**BF_{10}**	**Statistic**	***p***	**Effect size**	**BF_{10}**
Pre	w1	-0.28	0.609	-0.07	0.26	0.62	0.270	0.15	0.437
	w2	79.0	0.298	0.16	0.23	-2.28	0.981	-0.57	0.094
	w3	0.31	0.311	0.12	0.38	-1.49	0.923	-0.37	0.117
Sleep	w1	1.97	**0.063**	0.49	1.03	0.69	0.250	0.17	0.465
	w2	116.0	**0.005**	0.70	**2.38**	1.84	**0.042**	0.46	**1.912**
	w3	86.0	0.188	0.26	1.09	1.76	**0.049**	0.44	**1.703**
Post	w1	-0.77	0.774	-0.19	0.12	2.05	**0.029**	0.51	**2.602**
	w2	85.0	0.202	0.25	0.11	0.77	0.227	0.19	0.053
	w3	104.0	**0.033**	0.52	**2.30**	1.50	**0.076**	0.37	**1.195**

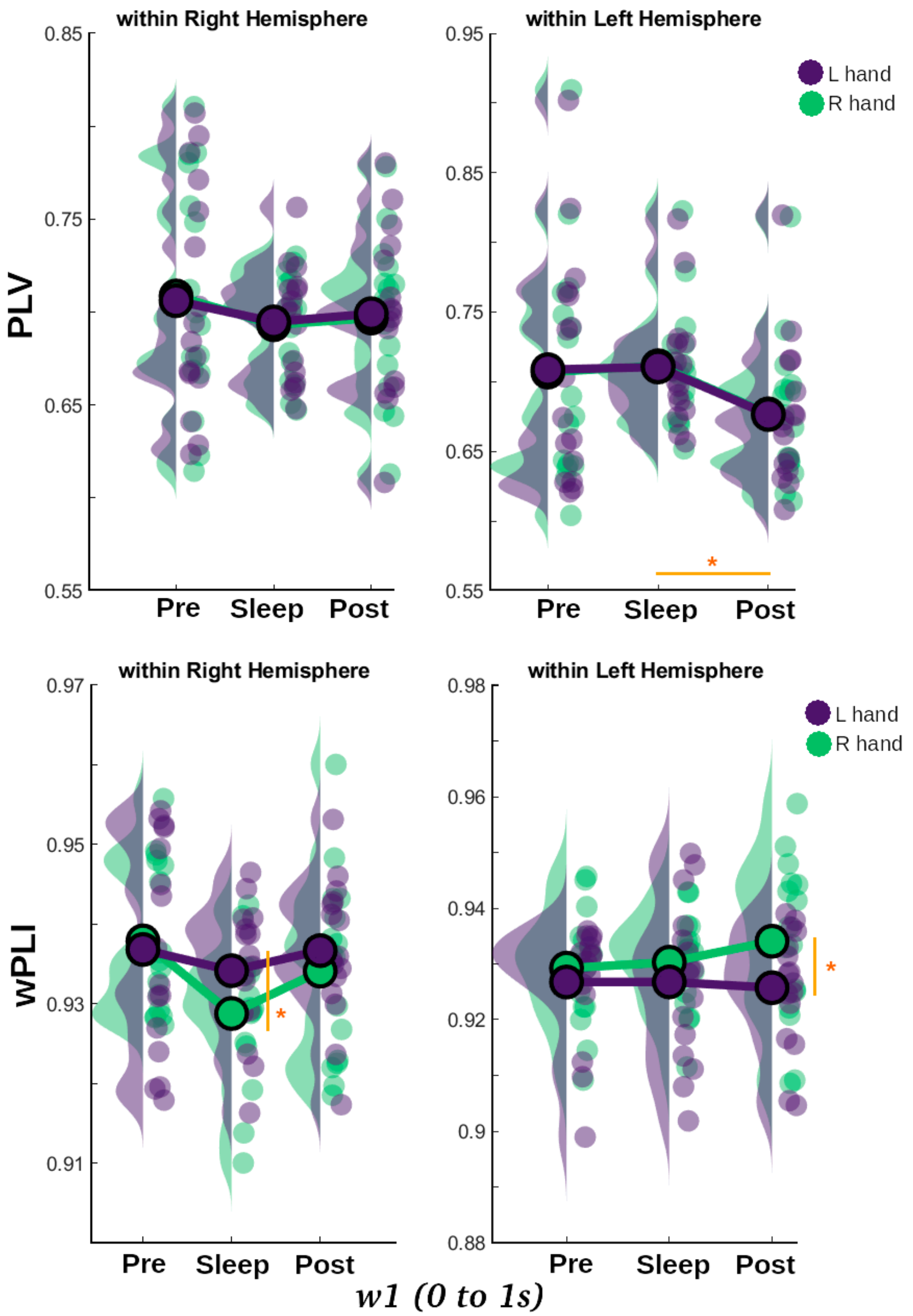

Figure S3. Repeated Measures Anova for PLV (upper row) and wPLI (bottom row) for within right hemisphere (left column) and within left hemisphere (right column) and w1. R hand is shown in green and L hand is represented in purple. Statistically significant results are highlighted in orange (p<0.05, ** p<0.01, ◊ p<0.09).*

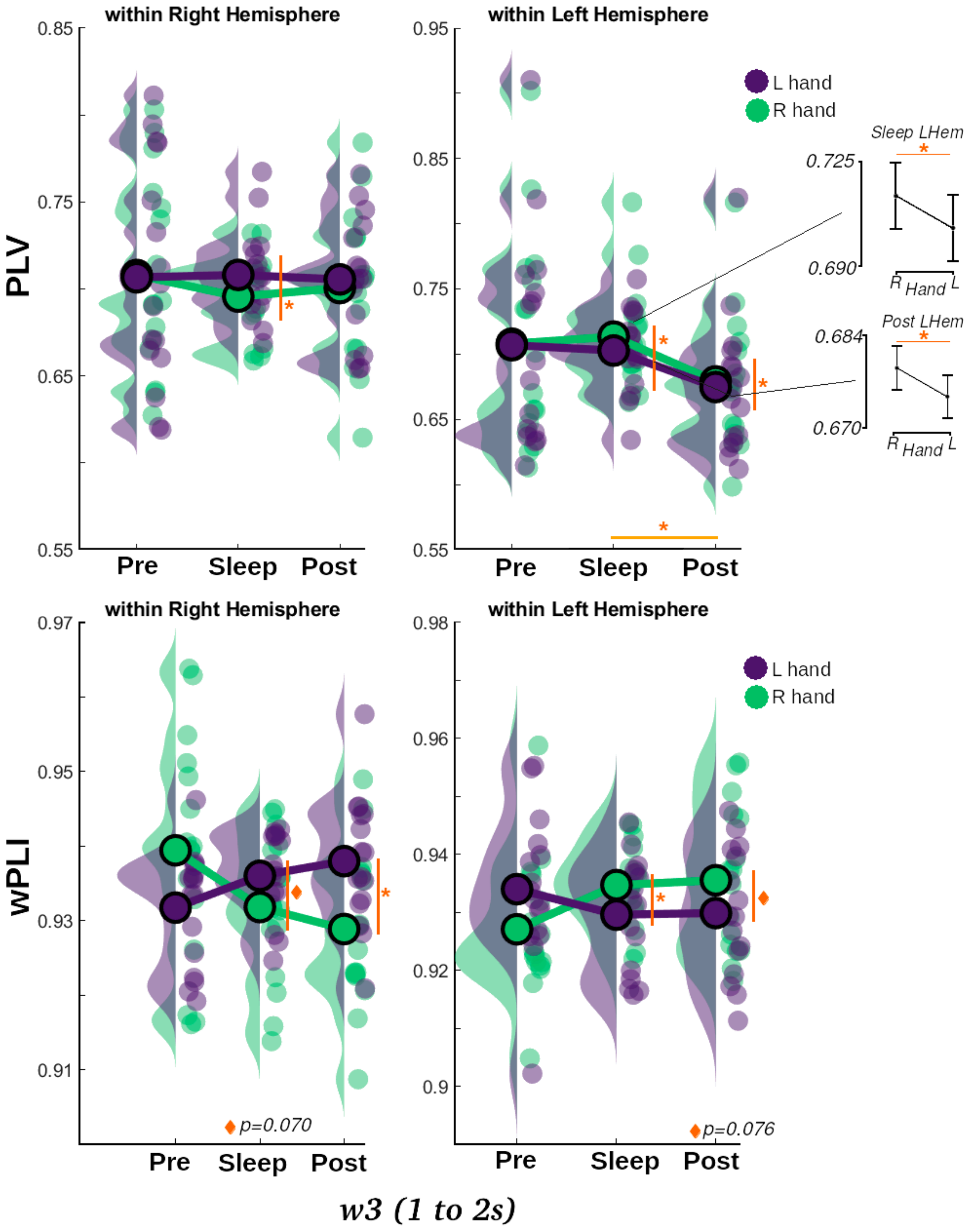

Figure S4. Repeated Measures Anova for PLV (upper row) and wPLI (bottom row) for within right hemisphere (left column) and within left hemisphere (right column) and w3. R hand is shown in green and L hand is represented in purple. Statistically significant results are highlighted in orange (p<0.05, ** p<0.01, ◊ p<0.09). Top-left corner graph have two zoomed*

Supplementary Materials S4: correlations

For each connectivity we calculated within right hemisphere (left column) and within left hemisphere (right column) values, that are also separated by R and L (the former in a yellowish background) hands. Correlation values are expressed by its value *Rho* and the bootstrapped (n=50000) corrected *p*-value. For clarity only correlations under 0.150 are depicted in the tables. Statistically significant correlations are highlighted in bold and darker background.

Table S15 Correlations between within hemispheres PLV-connectivity and behavioural measures.

PLV									
Behaviour	Session	**Within Right Hemisphere**				**Within Left Hemisphere**			
		**R hand**		**L hand**		**R hand**		**L hand**	
		Rho	*p*	Rho	*p*	Rho	*p*	Rho	*p*
**Reactivated blocks**									
sss pre									
sss post early	Sleep w1			0.54	0.116				
	**Sleep w2**			**0.63**	**0.037**				
sss post early									
ssi early	Sleep w1								
	Sleep w2			**0.63**	**0.057**				
	Post w1	-0.55	0.109						
	Post w2			-0.55	0.125				
	Post w3			-0.53	0.148				
ssi late									
**Non- Reactivated blocks**									
sss pre									
sss post early	Sleep w2			0.50	0.143				
sss post late									
ssi early	Post w2			-0.55	0.107				
	Post w1			-0.48	0.146				
ssi late	Pre w1			-0.55	0.108				
	Pre w3			-0.55	0.106				
	Sleep w2	-0.50	0.12					0.58	**0.066**

Table S16 Correlations between within hemispheres wPLI-connectivity and behavioural measures.

wPLI									
Behaviour	Session	Within Right Hemisphere				Within Left Hemisphere			
		R hand		L hand		R hand		L hand	
		Rho	*p*	Rho	*p*	Rho	*p*	Rho	*p*
Reactivated blocks									
sss pre	Sleep w3			0.47	0.159	-0.53	0.088		
	Post w3			0.47	0.159			0.47	0.129
sss post early									
sss post late	Sleep w3					-0.50	0.13		
ssi early	Sleep w2							-0.59	0.061
	Sleep w3	0.49	0.144						
ssi late	Sleep w3					-0.50	0.150		
Non-Reactivated blocks									
sss pre	Sleep w1					-0.66	0.053		
	Post w2	0.54	0.099						
sss post early	Sleep w2					-0.56	0.102		
	Sleep w3			0.50	0.130				
sss post late	Sleep w2					-0.54	0.114		
ssi early	Sleep w2					-0.51	0.136		
ssi late									

Supplementary Materials S5: Power analysis

All spectral analysis was performed in fieldtrip for each participant and window of interest without separate between L and R hand conditions. Power spectrum was calculated in the theta band (3-8Hz) using 3 cycles wavelets. The z-normalised values were then averaged within that frequency band and compared between sessions using cluster permutations Fieldtrip function (permutations=10000, alpha=0.05). There were not significant differences among sessions for any of the cases, with the lowest p-value (p=0.099) between Pre and Seep for w1 in the occipital channel 02.
